# PEN Receptor GPR83 in Anxiety-Like Behaviors: Differential Regulation in Global vs Amygdalar Knockdown

**DOI:** 10.3389/fnins.2021.675769

**Published:** 2021-08-26

**Authors:** Amanda K. Fakira, Lindsay M. Lueptow, Nikita A. Trimbake, Lakshmi A. Devi

**Affiliations:** Department of Pharmacological Sciences, Icahn School of Medicine at Mount Sinai, New York, NY, United States

**Keywords:** GIR, proSAAS, nucleus accumbens, stress, parvalbumin, dexamethasone, sex-differences, shRNA

## Abstract

Anxiety disorders are prevalent across the United States and result in a large personal and societal burden. Currently, numerous therapeutic and pharmaceutical treatment options exist. However, drugs to classical receptor targets have shown limited efficacy and often come with unpleasant side effects, highlighting the need to identify novel targets involved in the etiology and treatment of anxiety disorders. GPR83, a recently deorphanized receptor activated by the abundant neuropeptide PEN, has also been identified as a glucocorticoid regulated receptor (and named GIR) suggesting that this receptor may be involved in stress-responses that underlie anxiety. Consistent with this, GPR83 null mice have been found to be resistant to stress-induced anxiety. However, studies examining the role of GPR83 within specific brain regions or potential sex differences have been lacking. In this study, we investigate anxiety-related behaviors in male and female mice with global knockout and following local GPR83 knockdown in female mice. We find that a global knockdown of GPR83 has minimal impact on anxiety-like behaviors in female mice and a decrease in anxiety-related behaviors in male mice. In contrast, a local GPR83 knockdown in the basolateral amygdala leads to more anxiety-related behaviors in female mice. Local GPR83 knockdown in the central amygdala or nucleus accumbens (NAc) showed no significant effect on anxiety-related behaviors. Finally, dexamethasone administration leads to a significant decrease in receptor expression in the amygdala and NAc of female mice. Together, our studies uncover a significant, but divergent role for GPR83 in different brain regions in the regulation of anxiety-related behaviors, which is furthermore dependent on sex.

## Introduction

Anxiety disorders manifest in a variety of symptoms, but often involve excessive and/or persistent worry and fear that is considered maladaptive and intensifies over time. These disorders occur in approximately 20% of the population, and therefore represent a significant societal and economic burden. Anxiety disorders also occur at a higher rate in females compared to males (Palanza, 2001; Boivin et al., 2017; Zuloaga et al., 2020), and females respond differentially to anxiolytic drugs (Palanza, 2001) suggesting differences in the etiologies and/or underlying circuitry for anxiety between males and females. There are several neurotransmitter systems such as the γ-aminobutyric acid (GABA)-ergic and serotonergic systems, as well as neuropeptide systems including neuropeptide Y, cholecystokinin, corticotrophin releasing factor, and substance P that are targets for anxiety disorders (Griebel and Holmes, 2013; Murrough et al., 2015). Despite decades of continued research into the treatment of anxiety by targeting these major neurotransmitter systems, little progress has been made in the development of more efficacious treatments. This has led to a shift in research focus to other lesser known neurotransmitter and peptide systems with the intent of identifying the next generation of anxiolytics. One underutilized source of novel therapeutics is the pool of orphan G protein-coupled receptors (GPCRs) whose endogenous ligands are beginning to be explored.

Studies focusing on deorphanizing hypothalamic orphan GPCRs led to the identification of the abundant neuropeptide PEN as an endogenous ligand for the orphan receptor GPR83 (Gomes et al., 2016; Foster et al., 2019; Parobchak et al., 2020). The PEN peptide is a product of the cleavage of the proSAAS precursor (Fricker et al., 2000; Mzhavia et al., 2002), and has been implicated in a number of neurologic functions and disorders including feeding, reward, and Alzheimer’s disease (Wei et al., 2004; Wardman et al., 2011; Hoshino et al., 2014; Wang J. et al., 2016; Berezniuk et al., 2017). In fact, proSAAS and its peptide products have also been implicated in anxiety-related behavior (Wei et al., 2004; Morgan et al., 2010; Bobeck et al., 2017). Specifically for GPR83, it was found that the glucocorticoid receptor agonist, dexamethasone, regulates its expression in immune cells and the brain (Harrigan et al., 1989, 1991; Adams et al., 2003) suggesting that activation of stress-responses regulates GPR83 expression. Finally, a study examining behaviors of mice lacking GPR83 noted that they were resilient to stress-induced anxiety (Vollmer et al., 2013). These data have suggested a role for GPR83 in modulating anxiety-related behaviors (Lueptow et al., 2018; Mack et al., 2019).

In this study, we sought to directly investigate the role of GPR83 in anxiety-related behaviors, with a specific focus on female mice who have been previously overlooked, and furthermore, to determine the extent to which GPR83 expression in the amygdala subnuclei and nucleus accumbens (NAc) contribute to these behaviors. To date, no small molecule agonists or antagonists for this receptor have been identified, therefore we used a combination of GPR83 global knockout (KO) animals and GPR83 shRNA mediated local knockdown (KD) in the basolateral amygdala (BLA), central nucleus of the amygdala (CeA), and NAc to study the role of this receptor in anxiety-related behaviors. These brain regions play a role in anxiety, display significant GPR83 expression, and form circuits that encode positive and negative affective valence (Stuber et al., 2011; Tye et al., 2011; Janak and Tye, 2015; Namburi et al., 2015; Tovote et al., 2015; Beyeler et al., 2016; Lueptow et al., 2018; Fakira et al., 2019). Our initial behavioral studies also included both male and female subjects to determine whether GPR83 plays a differential role in anxiety-related behaviors between the two sexes. We found that the knockdown of GPR83 in the BLA of female mice resulted in increased anxiety-related behaviors and that dexamethasone administration led to sex-specific regulation of GPR83 expression supporting a role for this receptor in modulating anxiety-related behaviors which are dependent on sex.

## Materials and Methods

### Animals

GPR83/eGFP (Rockefeller University, New York, NY, United States), GPR83 KO and C57BL/6J (Jackson Labs, Bar Harbor, ME, United States) male and female mice (8–12 weeks) were maintained on a 12 h light/dark cycle with water and food *ad libitum*. GPR83/eGFP BAC transgenic mice were generated by the GENSAT project at Rockefeller University. The coding sequence for enhanced green fluorescent protein (eGFP) followed by a polyadenylation signal was inserted into a bacterial artificial chromosome (BAC) at the ATG transcription codon of GPR83. Therefore, cells that express GPR83 mRNA also express eGFP. GPR83 knockout (Jackson Labs, Bar Harbor, ME, United States) mice, generated previously (Lu et al., 2007), lack GPR83 protein and mRNA (Gomes et al., 2016; Fakira et al., 2019). Animal protocols were approved by the IACUC at Icahn School of Medicine at Mount Sinai, according to NIH’s Guide for the Care and Use of Laboratory Animals. The number of animals of each sex per group for each experiment is indicated on the individual figure legends.

### Elevated Plus Maze and Open Field Assay

One week prior to testing mice were handled for 5 min a day for 3–4 days. On the day of testing, mice were habituated to the testing room 1-h before open field testing followed by the elevated plus maze (EPM) 4 h later. In one of the cohorts the data for the open field assay was lost due to a computer issue therefore, in some cases the number of mice in the EPM analysis was greater than the open field assay. The open field consists of a 40 × 40 × 40 cm box made of white plastic material. Mice explored the open field for 30 min under red light illumination. The amount of time spent in the center of the open field was tracked for the first 5 min and the distance traveled was tracked for 20 min. The EPM consisted of two open and two closed arms (12 × 50 cm each) on a pedestal 60 cm above the floor. Mice explored the maze for 5 min under red light. The amount of time spent in each arm was tracked and analyzed using Noldus EthoVision XT. Data are presented as center time (s), distance traveled (cm), and open arm time [%; open arm time / (open arm + closed arm time)]. Vaginal swabs from the female mice were collected and visualized immediately following behavioral testing as described previously (McLean et al., 2012). Images were collected from the vaginal smears from each animal. The images were later visualized by two blinded investigators who categorized them as proestrus, estrous, metestrus, and diestrus by the presence of nucleated epithelial cells, cornified epithelial cells, and leukocytes. During metestrus and diestrus leukocytes predominate while, proestrus and estrus is characterized by nucleated and cornified epithelial cells. For analysis, female mice were grouped together as mice in estrus/proestrus, when follicle stimulating hormone, estradiol, luteinizing hormone, and prolactin levels are high. This group of mice are referred to as estrus throughout the article. The mice in diestrus and metestrus, when progesterone levels predominate, were also grouped together and are referred to as diestrus throughout the article (Miller and Takahashi, 2014).

### Immunofluorescence and Confocal Imaging

GPR83/eGFP mice were perfused with 4% paraformaldehyde in phosphate buffered saline pH 7.4. The brains were removed and post fixed in 4% paraformaldehyde in phosphate buffered saline overnight. Brains were rinsed three times in phosphate buffered saline and 50 μM coronal brain slices were obtained using a vibratome (Leica VT1000, Buffalo Grove, Il), without embedding the tissue. To visually enhance eGFP expression, immunohistochemical analysis was carried out using chicken anti-GFP (1:1000) as the primary antibody (Aves Labs, Tigard, OR, United States) and anti-chicken 488 (1:1000) as the secondary antibody (Molecular Probes, Eugene, OR, United States). In addition, brain slices were co-stained for parvalbumin (1:250; Thermo Fisher Scientific, Rockford, IL, United States) overnight followed by anti-sheep 568 (1:500) secondary antibody. Confocal microscopy was performed in the Microscopy CoRE at the Icahn School of Medicine at Mount Sinai. Confocal z-stack images were taken on a Zeiss LSM 780 microscope and processed using Zeiss software. Confocal images of the amygdala were taken from regions from coronal sections between Bregma −0.58 and −2.06 mm (Paxinos and Franklin, 2012).

### Dexamethasone Treatment and Quantitative PCR

Male and female mice were injected with dexamethasone (5 mg/kg; i.p.). Three hours later amygdala and NAc punches were collected for qPCR analysis. Total cellular RNA was extracted from amygdala and NAc punches using Qiazol reagent and the RNAeasy Midi kit (QIAGEN, Valencia, CA, United States). Total RNA was reverse transcribed into cDNA using VILO master mix (Invitrogen, Carlsbad, CA, United States). qPCR was performed in triplicate aliquots from each individual animal with Power SYBR Green PCR master mix (Thermo Fisher Scientific, Waltham, MA, United States), 25 ng of cDNA and 0.5 μM of primers using an ABI Prism 7900HT (Thermo Fisher Scientific, Waltham, MA, United States) in the qPCR CoRE at Icahn School of Medicine at Mount Sinai. Primer sequences for GPR83, proSAAS and GAPDH are the same as used previously (Fakira et al., 2019). The primer sequences used for qPCR are: GAPDH Forward: 5′-TGAAGGTCGGTGTGAACG Reverse: 5′-CAATCTCCACTTTGCCACTG, GPR83 Forward: 5′-GCAGTGAGATGCTTGGGTTC Reverse: 5′-CCCACCAAT AGTATGGCTCA, and proSAAS: Forward: 5′-AGTGTATG ATGATGGCCC Reverse: 5′-CCCTAGCAAGTACCTCAG. The CT values for the technical replicates were averaged and analysis performed using the ΔΔC_*T*_ method and normalized to saline controls. In some cases, qPCR reactions were repeated to determine the reliability of the primers and RNA samples.

### GPR83 shRNA and Surgeries

Three weeks prior to behavioral testing, a craniotomy was performed under isoflurane anesthesia and 0.5 μL of lentiviral GPR83 shRNA or control shRNA particles (10^9^; Sigma Mission Lentiviral Transduction Particles, St. Louis, MO, United States) were infused into the NAc (A/P: +1.5, Lat: ±1.6, D/V: −4.4), BLA (A/P: −1.1, Lat: ±3.2, D/V: −5.1), or CeA (A/P: −1.0, Lat: ±2.8, D/V: −4.9). The GPR83 shRNA targeted the sequence 5′-CCATGAGCAGTACTTGTTATA-3′, an exonic region of the gene. A nucleotide BLAST of this sequence produces three alignments with *E*-values of 0.003 that correspond to GPR83 variants; other alignments have *E*-values greater than 40, indicating that this sequence has few off targets.

### Data Analysis

Data are presented as mean ± SEM. Data were analyzed using unpaired *t*-test or two-way ANOVA with Tukey’s Multiple Comparison *post hoc* tests using GraphPad Prism 9.0 software (San Diego, CA, United States). The number of animals/group for each experiment is indicated on the individual figure legends.

## Results

### Analysis of Anxiety-Related Behaviors in Male and Female GPR83 KO Mice

**FIGURE 1:**
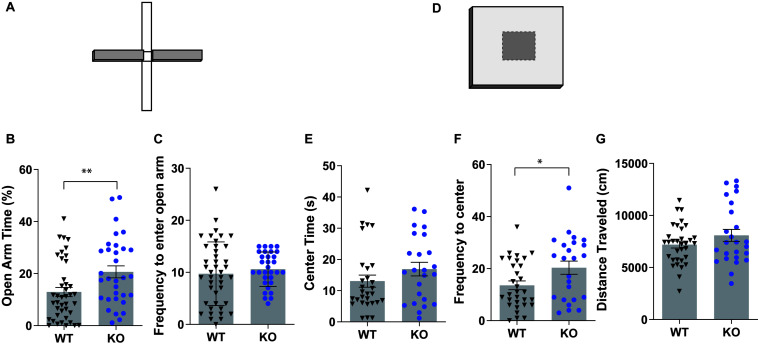
Mice lacking GPR83 have a decrease in anxiety-related behaviors. Wild-type (WT) and GPR83 knockout (KO) mice were screened on the elevated plus maze **(A)** and the amount of time spent on the open arms **(B)** and frequency to enter the open arms **(C)** measured. WT and GPR83 KO mice were screened in an open field assay **(D)** and the amount of time spent in the center **(E)**, frequency to enter to center **(F)** and distance traveled **(G)** measured. Data are represented as mean ± SEM and analyzed using unpaired *t*-test, % = open arm time/(open arm + closed arm time), *p < 0.05; **p < 0.01; WT, *n* = 41; GPR83 KO, *n* = 32.

Anxiety-related behaviors in GPR83 WT and KO mice were analyzed using the EPM (Figure 1A) and open field tests (Figure 1D). GPR83 KO mice spent more time in the open arms of the EPM compared to wild-type mice, indicating lower anxiety-like behavior in KO mice; however, there was no difference in the frequency to enter the open arms (unpaired *t*-test, open arm time, *t* = 2.704, df = 71; frequency, *t* = 0.7211, df = 71; Figures 1B,C). In the open field assay, there was no difference in the amount of time GPR83 KO mice spent in the center of the open field; however, there was a significant increase in the frequency that they entered the center region, also an indication of lower anxiety-like behavior (unpaired *t*-test, center time, *t* = 1.332, df = 51; frequency, *t* = 2.315, df = 53; Figures 1E,F). Overall, these differences are unlikely to be due to changes in overall motor activity of GPR83 KO mice, since there were no differences in locomotor activity in the open field (unpaired *t*-test, *t* = 1.399, df = 56; Figure 1G). Together, these results show that global loss of GPR83 produces a decrease in anxiety levels.

**FIGURE 2:**
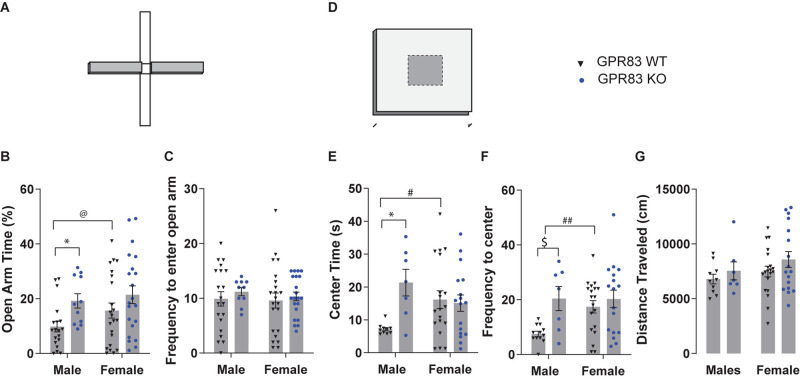
Sex-differences in GPR83-mediated regulation of anxiety-related behaviors. Sex-dependent analysis of WT and GPR83 KO mice on the elevated plus maze **(A)** measuring open arm time **(B)**, and frequency to enter the open arm **(C)**. Sex-dependent analysis of WT and GPR83 KO mice in the open field assay **(D)** measuring center time **(E)**, frequency to enter the center **(F)**, and distance traveled **(G)**. Data are represented as mean ± SEM and analyzed using two-way ANOVA following Tukey’s *post hoc* test (*p < 0.05) and unpaired *t*-test (^$^*p* = 0.07), % = open arm time/(open arm + closed arm time), ^@^*p* = 0.0872; ^#^*p* < 0.05, ^##^*p* < 0.01; WT males, *n* = 19, GPR83 KO males, *n* = 11; WT females, *n* = 22, GPR83 KO females, *n* = 21.

The data were further analyzed to determine the extent to which sex differences might contribute to anxiety-related behaviors in the global GPR83 KO mice. In the EPM test (Figure 2A), when separated by sex, GPR83 KO mice exhibited a significant effect on open arm time, with no interactions between sex and GPR83 KO genotype (Figure 2B; two-way ANOVA; interaction *F*_(__1_,_69__)_ = 0.38, *p* = 0.54; GPR83 KO *F*_(__1_,_69__)_ = 6.69, *p* = 0.012; sex *F*_(__1_,_69__)_ = 1.9, *p* = 0.17). In addition, Tukey’s *post hoc* analysis did not indicate any differences between groups; however, male GPR83 KO mice spent more time in the open arm of the EPM compared to wild-type when analyzed by unpaired *t*-test (Figure 2B; ^∗^*p* < 0.05). Neither analysis revealed any differences in the frequency to enter the open arm for either sex (Figure 2C; two-way ANOVA; interaction *F*_(__1_,_69__)_ = 0.047, *p* = 0.83; GPR83 genotype *F*_(__1_,_69__)_ = 0.64, *p* = 0.47; sex *F*_(__1_,_69__)_ = 0.21, *p* = 0.65). Direct comparison of open arm time in wild-type males and females indicates that there is a tendency for female mice to spend more time in the open arm compared to males (Figure 2B; unpaired *t*-test ^@^*p* = 0.0872, *t* = 1.751, df = 42; Tukey’s *post hoc p* = 0.42), indicating that female mice may be less anxious compared to males overall.

In the open field test (Figure 2D) the sex-dependent analysis of anxiety-related behavior revealed that male GPR83 KO mice spent significantly more time in the center compared to wild-type males, an effect that was not seen when comparing female GPR83 KO mice with wild-type females (Figure 2E; two-way ANOVA; interaction *F*_(__1_,_49__)_ = 6.34, *p* = 0.0150; GPR83 KO *F*_(__1_,_49__)_ = 4.67, *p* = 0.0355; sex *F*_(__1_,_49__)_ = 0.15, *p* = 0.7044; Tukey’s *post hoc* test males: WT vs GPR83 KO, ^∗^*p* < 0.05). Moreover, analysis of frequency to enter the center indicates a similar effect in that male GPR83 KO mice entered the center of the open field more frequently than male wild-type mice while no differences were seen between female GPR83 KO mice and female wild-type mice (Figure 2F; two-way ANOVA; interaction *F*_(__1_,_49__)_ = 2.53, *p* = 0.1175; GPR83 *F*_(__1_,_49__)_ = 6.36, *p* = 0.0.015; sex *F*_(__1_,_49__)_ = 2.4, *p* = 0.13; Tukey’s *post hoc* test males: WT vs GPR83 KO, ^$^*p* = 0.07). Analysis of baseline anxiety differences between wild-type male and female mice indicate that female mice display significantly less anxiety-related behaviors levels, spending more time in the center (^#^*p* < 0.05, unpaired *t*-test, *t* = 2.350, df = 29; Tukey’s *post hoc p* = 0.11) and entering the center of the open field more frequently (^##^*p* < 0.01 unpaired *t*-test, *t* = 3.094, df = 27; Tukey’s *post hoc p* = 0.054) than males (Figures 2E,F). In addition, we find no effect of GPR83 KO on locomotor activity levels even when segregated by sex (Figure 2G; two-way ANOVA; interaction *F*_(__1_,_50__)_ = 0.06, *p* = 0.8036; GPR83 *F*_(__1_,_50__)_ = 1.754, *p* = 0.19; sex *F*_(__1_,_50__)_ = 1.52, *p* = 0.22). Overall, these data indicate that lack of GPR83 produces a decrease in anxiety-related behaviors that is more pronounced in male mice, likely due to their higher levels of baseline anxiety compared to female mice.

### Cell-Type Specific Expression of GPR83 in the Amygdala

**FIGURE 3:**
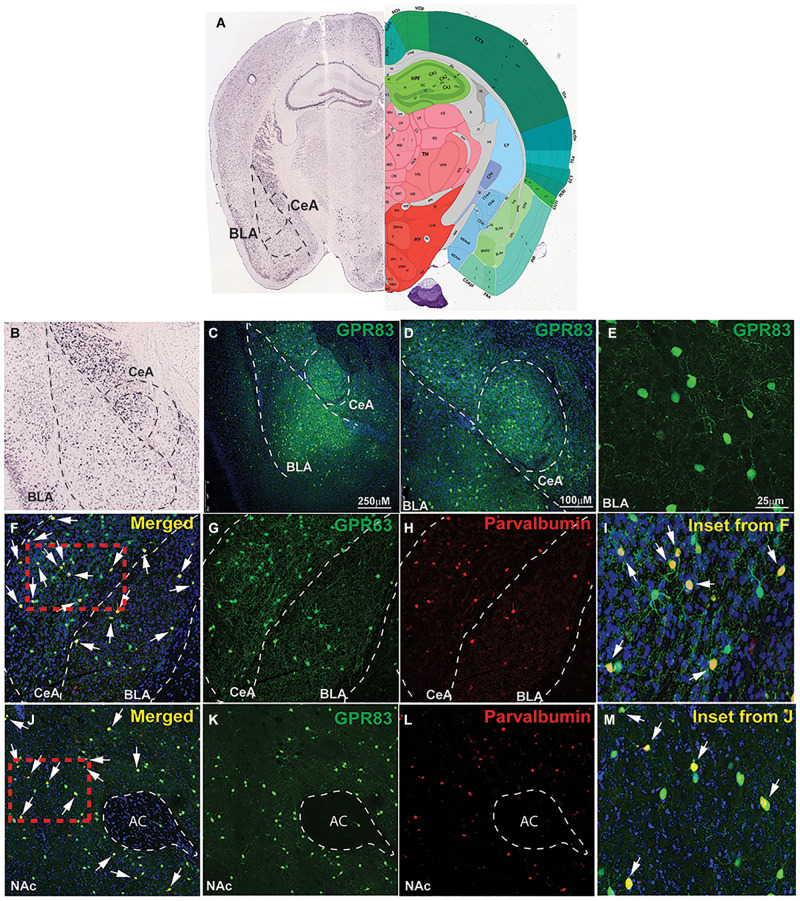
GPR83 expression in the basolateral and central nucleus of the amygdala. **(A)** *In situ* hybridization image for GPR83 from the Allen Mouse Brain Atlas (Allen Institute. 2015 Allen Institute for Brain Science. Allen Brain Atlas API) (left) and corresponding brain atlas image (right). **(B)** Enlarged image of BLA and CeA shown in **(A)**. Low magnification image of GPR83 (green) expression in the BLA and CeA **(C,D)** using GPR83-GFP reporter mice from Gensat. **(E)** Higher magnification image showing GPR83 expression in neurons in the amygdala. **(F)** Co-localization (yellow, arrows) of GPR83 (green, **G**) and parvalbumin (red, **H**) in the BLA and CeA. **(I)** Inset from **F**. **(J)** Co-localization (yellow, arrows) of GPR83 (green, **K**) and parvalbumin (red, **L**) in the NAc. AC, anterior commissure. **(M)** Inset from **J**.

The amygdala is a brain region well known to play a role in anxiety-related behaviors (Gilpin et al., 2015; Janak and Tye, 2015; Tovote et al., 2015). Within the BLA, parvalbumin cells (PV^+^) are the largest population of GABAergic inhibitory interneurons (McDonald and Mascagni, 2001), directly influencing output of primary excitatory neurons, and these cells have been directly implicated in anxiety-like behavior (Urakawa et al., 2013; Babaev et al., 2018). In addition, PV^+^ neurons within the central amygdala (CeA), have been implicated in anxiety trait (Ravenelle et al., 2014), as well as opioid withdrawal-induced negative affect, including anxiety-like behavior (Wang L. et al., 2016). *In situ* hybridization data from the Allen Mouse Brain Atlas indicates that GPR83 is expressed in both the BLA and CeA (Figures 3A,B). Therefore, we sought to examine the co-expression of PV^+^ and GPR83^+^ cells in this brain region using GPR83/GFP BAC transgenic mice. These mice express eGFP under control of the GPR83 promotor; therefore all cells that express the receptor will also express eGFP. We find that GPR83 is expressed throughout the amygdala (Figure 3C) with higher expression in the BLA and the CeA (Figures 3C,D). Higher magnification images demonstrate that the eGFP positive cells have a neuronal morphology (Figure 3E). Subsequent co-staining with parvalbumin indicates that some of the GPR83 positive cells express parvalbumin and, therefore are GABAergic neurons (Figures 3F–I). Not all of the GPR83 positive neurons co-localize with parvalbumin. It is possible that these GPR83 positive and parvalbumin negative neurons are somatostatin neurons. This possibility will be investigated in future studies characterizing the identity of GPR83 neurons in the central nervous system.

The nucleus accumbens is another brain region that contains a high concentration of GPR83 positive cells, and has been strongly implicated in vulnerability and resilience responses to stress (Zhu et al., 2017) as well as anxiety-like behaviors (Xiao et al., 2020). In contrast to the amygdala, we have recently reported that GPR83 is primarily expressed in cholinergic interneurons in the NAc (Fakira et al., 2019). However, a small percentage of neurons were not characterized but recent studies demonstrated that PV^+^ neurons in the striatum indeed express GPR83 (Enterría-Morales et al., 2020). Because PV^+^ neurons in the NAc have been specifically implicated in anxiety-like approach behaviors, we also characterized PV^+^ and GPR83^+^ co-expression in the NAc. We find that a small population of GPR83 positive cells in the NAc also express parvalbumin suggesting the presence of this receptor in some GABAergic neurons (Figures 3J–M).

### Divergent Regulation of GPR83 in Male and Female Mice Following Acute Dexamethasone Administration

**FIGURE 4:**
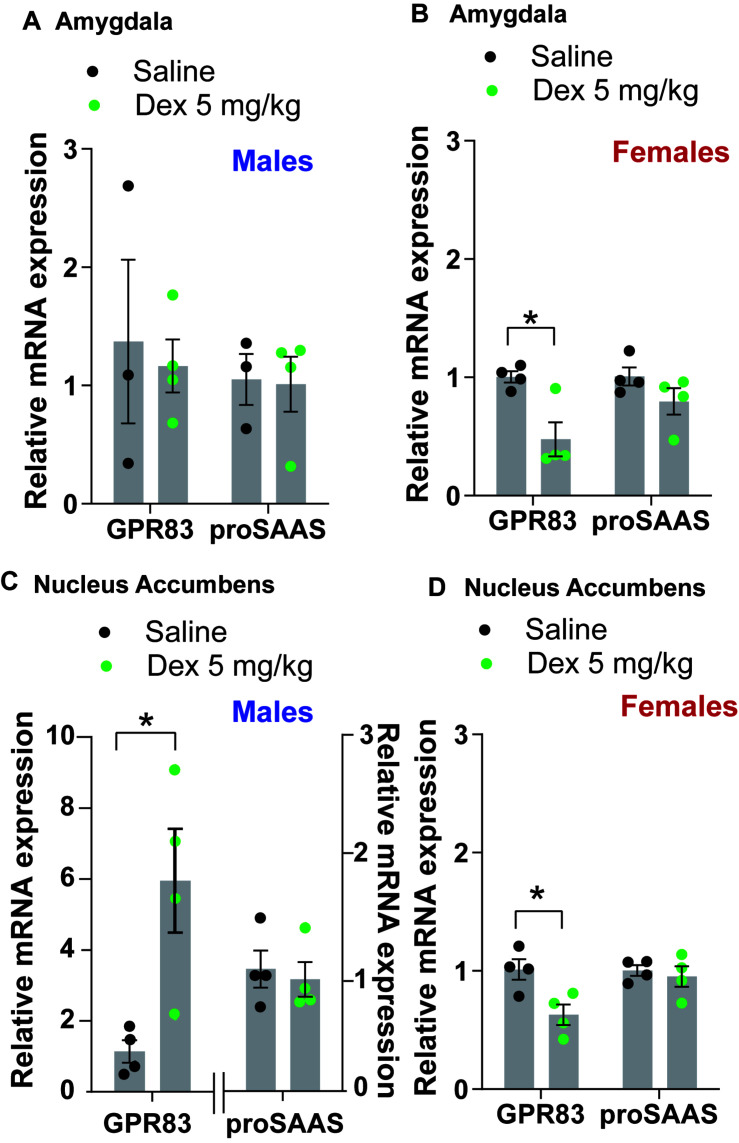
GPR83 expression is regulated by dexamethasone in the amygdala and nucleus accumbens in a sex-dependent manner. **(A,B)** Administration of dexamethasone (5 mg/kg) decreases expression of GPR83 in the amygdala of female mice but not males with no effects on proSAAS expression. **(C,D)** In the NAc, administration of dexamethasone increases expression of GPR83 in males and decreases the expression in females. The proSAAS expression is unchanged in all cases. Data are represented as mean ± SEM and analyzed using unpaired *t*-test, *p < 0.05, *n* = 3–4 per group.

Studies have shown that GPR83 expression is regulated by the glucocorticoid agonist dexamethasone (Harrigan et al., 1989; Adams et al., 2003) suggesting a role for GPR83 in the stress response. Because of this known association with the glucocorticoid system, we used a single dose of dexamethasone to assess its effects on GPR83 expression, in both amygdala and the NAc of male and female mice. As a control, we examined proSAAS expression, since proSAAS is the precursor to the endogenous ligand for GPR83, PEN. We find that although dexamethasone administration has no effect on GPR83 expression in the amygdala of male mice (unpaired *t*-test, *t* = 3.491, df = 6, *p* = 0.76) and a decrease in expression in female mice (females unpaired *t*-test, *t* = 3.491, df = 6, ^∗^*p* < 0.05; Figures 4A,B). In this experiment, GPR83 expression in the amygdala of the saline-treated male mice has a large variation between the three mice analyzed. In contrast, in the NAc, dexamethasone administration leads to an increase in GPR83 expression in male mice (unpaired *t*-test, *t* = 3.216, df = 6, ^∗^*p* < 0.05) and a decrease in expression in female mice (female unpaired *t*-test, *t* = 3.048, df = 5, ^∗^*p* < 0.05; Figures 4C,D). In this experiment, GPR83 expression in the NAc of saline-treated male mice does not have large variations between the four mice analyzed. ProSAAS expression was unchanged by dexamethasone administration in either sex or two brain regions tested (unpaired *t*-test, females amygdala, *t* = 1.561, df = 6, *p* = 0.17; females NAc, *t* = 0.5150, df = 6, *p* = 0.63; males amygdala, *t* = 0.1196, df = 5, *p* = 0.91; males NAc, *t* = 0.4124, df = 6, *p* = 0.69; Figures 4A–D). These results suggest that GPR83 expression is regulated by glucocorticoids in a region-specific and sex-dependent manner.

### The Effect of GPR83 Knockdown in the BLA, CeA, and NAc on Anxiety-Related Behaviors

**FIGURE 5:**
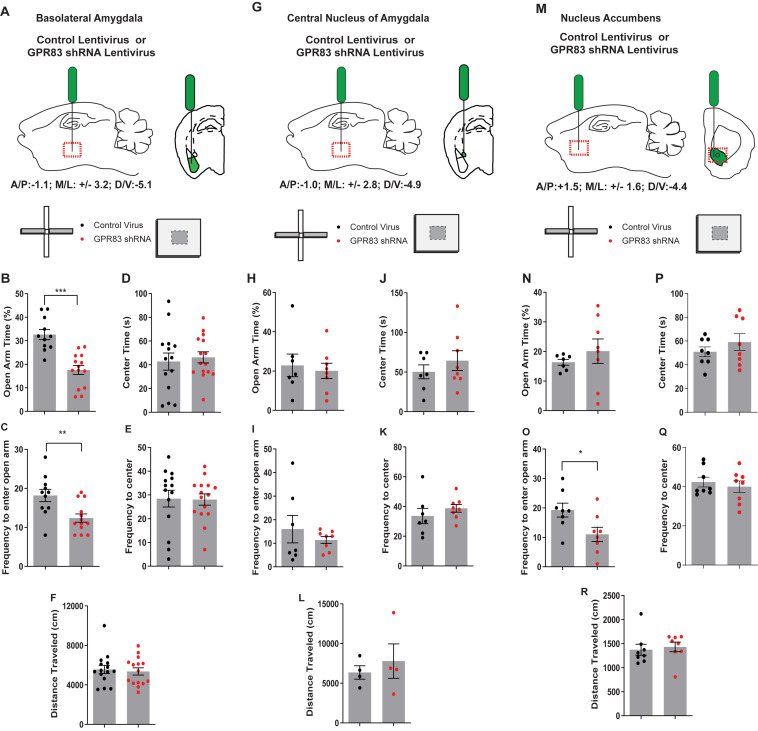
GPR83 knockdown in the BLA, but not the CeA or NAc increases anxiety-related behaviors in female mice. Schematic of injection of control or GPR83 shRNA lentivirus into the **(A)** BLA, **(G)** CeA, or **(M)** nucleus accumbens. Effect of brain region specific GPR83 knockdown in mice on open arm time in the elevated plus maze **(B,H,N)** and on the frequency to enter the open arm **(C,I,O)**. Effect of brain region specific GPR83 knockdown in mice on the center time in the open field assay **(D,J,P)**, on frequency to enter the center **(E,K,Q)** and on the distance traveled **(F,L,R)**. Data are represented as mean ± SEM and analyzed using unpaired *t*-test, % = open arm time/(open arm + closed arm time), *p < 0.05, **p < 0.01, ***p < 0.001, BLA, CV *n* = 11, GPR83 shRNA *n* = 14; CeA, CV *n* = 7, GPR83 shRNA *n* = 8; NAc, CV *n* = 8, GPR84 shRNA *n* = 8.

We observed that global GPR83 KO decreased anxiety-related behaviors in male mice but had limited effects on anxiety-related behaviors in females. Since female mice had higher baseline levels of anxiety compared to males, we sought out to determine whether a focused decrease in GPR83 expression in specific brain regions of female mice may induce a greater effect on anxiety-related behaviors. Since glucocorticoids, which are typically released during stressful events that produce anxiety, induce a decrease in GPR83 expression in the amygdala and NAc of female mice we sought to determine the effect of local GPR83 knockdown (GPR83 KD) in the BLA, CeA, and NAc on anxiety-related behaviors in female mice. The knockdown of GPR83 expression was accomplished by administration of GPR83 shRNA lentiviral particles (0.5 μl; 10^9^ particles/μl) into the BLA, CeA, or NAc of female mice (Figures 5A,G,M) and anxiety-related behaviors analyzed using the EPM and open field tests and compared to mice that were administered with control virus. In a previous study we showed that this paradigm of lentiviral GPR83 shRNA administration produces a ∼50% knockdown compared to control virus (Fakira et al., 2019). We find that local GPR83 KD in the BLA resulted in a decrease in the amount of time spent (unpaired *t*-test, *t* = 5.223, df = 23, ^∗∗∗^*p* < 0.001) and frequency to enter (unpaired *t*-test, *t* = 3.119, df = 22, ^∗∗^*p* < 0.01) the open arm of the EPM indicating an increase in anxiety-related behaviors (Figures 5B,C). However, these animals did not exhibit anxiety behaviors in the open field assay or overall locomotor activity (unpaired *t*-test, center time *x*, *t* = 0.4131, df = 27, *p* = 0.68; frequency, *t* = 0.08650, df = 27, *p* = 0.93; locomotor activity, *t* = 0.3624, df = 28, *p* = 0.72; Figures 5D–F). GPR83 KD in the CeA or NAc had no effect on these behaviors except for a decrease in the frequency to enter the open arm of the EPM in the case of the NAc (unpaired *t*-test, CeA, EPM, open arm time, *t* = 0.4106, df = 13, *p* = 0.69, frequency, *t* = 0.8184, df = 13, *p* = 0.43; open field, center time, *t* = 0.8946, df = 1, *p* = 0.34; frequency, *t* = 0.9299, df = 13, *p* = 0.37; locomotor activity, *t* = 0.6160, df = 6, *p* = 0.56; NAc EPM, open arm time, *t* = 0.8236, df = 13, *p* = 0.43, frequency, *t* = 2.441, df = 14, ^∗^*p* < 0.05; open field, center time, *t* = 0.9896, df = 14, *p* = 0.34, frequency, *t* = 0.6068, df = 14, *p* = 0.55, locomotor activity, *t* = 0.5735, df = 14, *p* = 0.58; Figures 5G–R). Overall, these data indicate that GPR83 expression in the BLA regulates anxiety levels in female mice however, revealing these differences depends on the sensitivity of the assay used.

The baseline anxiety levels in the control virus BLA injected animals (Figure 5B) is significantly greater than that of the control virus CeA and NAc injected mice (Figures 5H,N). The first two rounds of CeA and BLA injections were run side-by-side from injections to behavioral analysis. This elevated baseline level of anxiety was observed following these two rounds of experiments when the number of mice per group were equal between the CeA and BLA. In a separate set of control virus injections into the NAc, we observed the same baseline anxiety as observed in CeA injected mice. This leads us to believe that our injections into the BLA are having an impact on baseline anxiety levels. Due to the lack of observed differences with 7–8 mice per group in the CeA (*p* = 0.6881) and NAc (*p* = 0.4250) experiments we subsequently increased the number of BLA injected mice to confirm our effects as well as increase the number of mice in our estrous cycle comparison described below.

**FIGURE 6:**
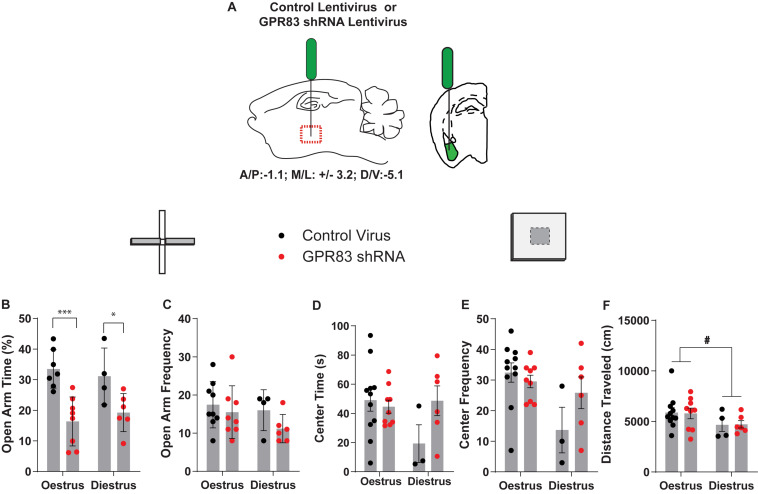
Analysis of estrus cycle-dependent differences in anxiety-related behaviors following GPR83 knockdown in the BLA. **(A)** Schematic of injection of control or GPR83 shRNA lentivirus into the BLA. Analysis of estrus cycle-dependent differences following GPR83 knockdown in the BLA, on the elevated plus maze and open field assays, measuring open arm time **(B)**, frequency to enter the open arm **(C),** center time **(D)**, frequency to enter the center **(E)**, and distance traveled **(F)**. Data are represented as mean ± SEM and analyzed using two-way ANOVA following Tukey’s *post hoc* test, % = open arm time/(open arm + closed arm time), *p < 0.05, ***p < 0.001, unpaired *t*-test, ^#^*p* < 0.05; *n* = 3–11 per group.

Next, we examined the estrus cycle-dependency on anxiety following GPR83 KD in the BLA (Figure 6A). For this, we monitored the estrus cycle by taking vaginal swabs immediately following behavioral testing which were categorized by two blind observers into the different stages by the presence of leukocytes, nucleated and cornified epithelial cells. Estrus was defined as mice in the proestrus and estrus phase during which circulating hormone levels peak. Diestrus was defined as mice in metestrus and diestrus during which circulating hormone levels are lower (Wood et al., 2007; Miller and Takahashi, 2014). This analysis revealed that both estrus and diestrus mice with GPR83 KD in the BLA exhibit significant decreases in time spent in the open arms indicating that the increases in anxiety are not estrus cycle-dependent (Figure 6B; two-way ANOVA; interaction *F*_(__1_,_21__)_ = 0.75, *p* = 0.3955; GPR83 KD *F*_(__1_,_21__)_ = 22.83, *p* = 0.0.0001; estrus cycle *F*_(__1_,_21__)_ = 0.01, *p* = 0.9314; Bonferroni *post hoc* test, estrus control virus vs GPR83 KD, *p* < 0.0001; diestrus control virus vs GPR83 KD *p* < 0.05). There was a trend for amygdala GPR83 KD mice in diestrus to enter the open arm less frequently than mice in estrus (Figure 6C; two-way ANOVA; interaction *F*_(__1_,_23__)_ = 0.38, *p* = 0.5460; GPR83 KD *F*_(__1_,_23__)_ = 2.07, *p* = 0.1649; estrus cycle *F*_(__1_,_23__)_ = 1.50, *p* = 0.2328; unpaired test, diestrus control virus vs GPR83 shRNA, *p* < 0.1272). Finally, further analysis of estrus cycle effects reveals a possible effect of estrus cycle on center time and frequency to enter the center which may be explained by an overall decrease in activity of mice in diestrus revealed by decreases in locomotor activity (Figures 6D–F; two-way ANOVA; interaction *F*_(__1_,_26__)_ = 0.02, *p* = 0.9023; GPR83 KD *F*_(__1_,_26__)_ = 0.00, *p* = 0.9668; estrus cycle *F*_(__1_,_26__)_ = 3.94, *p* = 0.0577; unpaired test estrus vs diestrus, *t* = 0.4228, df = 24, ^#^*p* < 0.05). Together, these data indicate that there is no difference in anxiety behaviors between mice in estrus vs diestrus however, diestrus decreases the overall activity levels of mice.

## Discussion

Early studies have shown that GPR83 expression is regulated by the glucocorticoid receptor agonist dexamethasone (Harrigan et al., 1989; Adams et al., 2003), suggesting a role for GPR83 in stress and anxiety responses, since glucocorticoid release is a hallmark of the stress response. In fact, studies have reported that mice lacking GPR83 are resistant to stress-induced anxiety (Vollmer et al., 2013). However, these studies did not examine sex-differences or the specific brain regions where GPR83-mediated regulation of anxiety-related behavior may occur.

We found that global loss of GPR83 leads to a decrease in anxiety-related behaviors which is more prominent in male compared to female mice. In agreement with other studies (Simpson et al., 2012), we found that female wild-type mice tend to display lower baseline levels of anxiety; this could account for the lack of effect of the global GPR83 KO on anxiety-related behaviors in female mice. In fact, these studies found that female mice were resistant to treatment with the anti-anxiety drug, diazepam, as compared to males, likely due to a floor effect in the female mice (Simpson et al., 2012). These data support the concept that lower baseline levels of anxiety in female mice may be a significant factor in screening treatments for anxiety. Together these data highlight the importance of examining the effectiveness of anxiety treatments on both males and females in preclinical models using multiple behavioral assays of anxiety, since specific assays may not be ideal for both sexes. In this context, future in-depth characterization of the role of GPR83 in anxiety will require screening in alternate assays besides the ones described in this study (EPM and open field), such as novelty suppressed feeding, marble burying, etc., in order to fully understand the role of this receptor system in modulating nuances of anxiety behaviors.

There are several classes of drugs for the treatment of anxiety disorders including those that act to control the balance between GABA and glutamate transmission (Murrough et al., 2015). The BLA contains local inhibitory neurons that regulate excitatory projections to the CeA, ventral hippocampus (vHPC), medial prefrontal cortex (mPFC), bed nucleus of the stria terminalis (BNST), and NAc (Sah et al., 2003; Janak and Tye, 2015; Tovote et al., 2015). While activation of the BLA projection to the mPFC and vHPC induces anxiety-related behaviors, activation of BLA projection to the CeA and BNST results in anxiolysis (Nascimento Häckl and Carobrez, 2007; Tye et al., 2011; Felix-Ortiz et al., 2013, 2016; Kim et al., 2013; Felix-Ortiz and Tye, 2014; Lowery-Gionta et al., 2018). Moreover, the circuits from the BLA to NAc and the BLA to CeA have been shown to encode positive and negative valence, respectively (Stuber et al., 2011; Namburi et al., 2015; Beyeler et al., 2016), suggesting that a complex network of circuits contribute to the overall anxiety state.

In our studies, complete removal of GPR83 in the knockout animal produced decreases in anxiety-related behaviors while specific knockdown in the BLA resulted in more anxiety-related behaviors. One reason for this discrepancy between the effect of global knockout vs local knockdown in the BLA may be due to an imbalance in these outgoing circuitries, suggesting the GPR83 tone from BLA contributes more to the anxiolysis, since there is an increase in anxiety with loss of GPR83 in this region, while global KO of GPR83 expression offsets this change in amygdalar tone. Previous studies have detected GPR83 expression in the PFC, hypothalamus, NAc, hippocampus, and BNST (Pesini et al., 1998; Brezillon et al., 2001; Wang et al., 2001; Eberwine and Bartfai, 2011; Dubins et al., 2012; Müller et al., 2013; Lueptow et al., 2018; Fakira et al., 2019), though the role of GPR83 in each of these brain regions has yet to be explored. The current studies suggest that removing GPR83 from all these regions may shift the overall output in a direction which favors less anxiety and the mechanisms that underlie this remains to be examined.

Though reducing expression of GPR83 in the BLA uncovered a shift toward increasing anxiety-related behaviors, GPR83 KD in the CeA and NAc had little to no effect. Previous studies showed that blocking excitatory output from the BLA to the CeA or mPFC resulted in a shift toward increasing anxiety-related behaviors (Tye et al., 2011; Felix-Ortiz et al., 2016; Lowery-Gionta et al., 2018). Therefore, it is possible that GPR83 expression on interneurons in the BLA regulates inhibitory control. In line with this concept, our studies identified GPR83 expression on parvalbumin positive GABAergic neurons, which are known to form perisomatic synapses, i.e., along the soma, axon initial segment, and proximal dendrites, of excitatory pyramidal neurons in the BLA representing half of their inhibitory input (McDonald and Mascagni, 2001; Muller et al., 2006). Therefore, these parvalbumin expressing neurons are in a prime position to gate output from the BLA. Furthermore, recent studies demonstrated that suppressing parvalbumin neuron activity in the BLA upregulates anxiety-related behaviors (Luo et al., 2020) similar to the increases in anxiety seen following GPR83 knockdown in the BLA. This suggests that reducing GPR83 expression on parvalbumin neurons may suppress parvalbumin neuron activity thereby resulting in a net increase in excitatory output to downstream brain regions. GPR83 did not completely colocalize with parvalbumin however, due to the pattern of staining and colocalization with interneurons in the amygdala and NAc it is possible that some of the GPR83 neurons are somatostatin positive. This concept will be explored in future studies. In order to determine the role of GPR83 on excitatory circuits in the BLA, future studies investigating the impact of GPR83 knockdown on inhibitory and excitatory neurotransmission are necessary.

Because GPR83 has been implicated in anxiety-related behaviors, and subcellular regulation of the receptor is altered by stress-related glucocorticoids, we investigated the impact of the glucocorticoid agonist, dexamethasone, on regulation of GPR83 expression in a sex and brain region specific manner. In the amygdala, we find that dexamethasone treatment reduced GPR83 expression in female mice but had no effect in males. While this is consistent with a previous study using male mice which reported that GPR83 expression in the amygdala was not affected by dexamethasone treatment (Adams et al., 2003), our study revealed a significant variability of GPR83 expression in the amygdala of male mice. This variability limits our interpretation of whether there is truly no effect of dexamethasone on GPR83 expression in the amygdala of male mice. In the NAc, we find that dexamethasone induced opposing effects on GPR83 expression, increasing expression in males while decreasing expression in females. Our observations with male mice are in contrast to those of Adams et al. (2003) that reported decrease in GPR83 expression in NAc following dexamethasone treatment in male mice. This discrepancy could be due to a number of factors including the strain of mice used (C57Bl6 vs ICR), sensitivity of the technique used (*in situ* hybridization vs real-time qPCR), and/or the effects of estrus cycle hormones in the female mice.

We have also found variability in GPR83 expression in individual male mice. This is particularly evident in the amygdala of saline treated male mice. This variability limits our interpretation of the effect of dexamethasone on GPR83 expression in the amygdala. It is known that the levels of corticosterone, the naturally occurring glucocorticoid in rodents, are higher in females compared to males (Nguyen et al., 2020). Based on this, female mice may have more stable expression of glucocorticoid regulated proteins such as GPR83 which may explain why female mice have less variable GPR83 expression. Additionally, this may explain why the effect of dexamethasone on GPR83 was more consistent between brain regions in females, where we observed a dexamethasone-induced decrease in both the NAc and amygdala. The higher levels of corticosterone in females may also contribute to the sex-differences in baseline anxiety and GPR83-mediated regulation of anxiety levels reported above. Overall, these studies highlight the need for further examination of the relationship between sex, glucocorticoid signaling, and GPR83 expression to fully explain the observed sex-differences and variability in GPR83 expression between the sexes.

Another important finding of this study is that knockdown of GPR83 in the BLA of female mice increased anxiety-related behaviors in the EPM test irrespective of whether the animals were in the estrus or diestrus stage. Our study was designed to limit stress exposure therefore, we collected vaginal swabs following behavioral tests. Due to this we were unable to control what stage in the estrous cycles the mice were in at the time of testing. Due to this fact, some of our groups have smaller numbers of mice, in particular, there only three to four mice in the control virus group in the diestrus phase during our behavioral testing. While we still saw a significant effect of GPR83 shRNA virus on anxiety-related behaviors in the EPM, the lower number of animals in diestrus group limits our interpretation, especially in the open field tests where it appears like mice in diestrus may have increases in anxiety-related behaviors but since the diestrus group is underpowered we cannot draw a conclusion.

A complete study of the effect of estrous cycle on GPR83 and its role in anxiety-related behaviors is warranted due to the potential observed in the current results. In addition, GPR83 expression in the uterus is regulated during the estrus cycle in an estrogen and progesterone dependent manner (Parobchak et al., 2020) suggesting that circulating hormone levels may influence GPR83 function. Studies have shown that females in proestrus display decreased anxiety-related behaviors which corresponded with higher levels of progesterone and its metabolite 5α-pregnan-3α-ol-20-one (3α-5α-THP; allopregnanolone) (Frye et al., 2000). In line with this, treatment of ovariectomized rats with progesterone is anxiolytic and corresponded with the potentiation of GABA_*A*_ receptor currents (Gulinello and Smith, 2003). Subsequent studies found that administration of allopregnanolone is anxiolytic when administered acutely. However, chronic allopregnanolone treatment is anxiogenic in both male and female mice and alters the anxiolytic potential of the benzodiazepine ligands lorazepam and flumazenil (Gulinello and Smith, 2003).

Our studies did not identify any differences in anxiety between wild-type females in estrus vs diestrus. This may be because in our studies we had pooled mice in groups with high circulating hormones (estrus-proestrus/estrus) and low circulating hormones (diestrus-metestrus/diestrus) (Miller and Takahashi, 2014). By pooling together animals with varying levels of individual hormones, these differences in anxiety levels may have reached below detectable threshold. Given the role of progesterone/allopregnanolone in modulating anxiety-related behaviors *via* regulation of GABAergic function, and that GPR83 expression is regulated by estrogen and progesterone in the uterus (Parobchak et al., 2020) further studies are needed to determine if there is a relationship between GPR83 and progesterone levels in the brain.

## conclusion

In summary, our studies suggest that GPR83 is differentially regulated between male and female mice. Furthermore, regional changes in expression of GPR83 significantly impacts the overall tone of anxiety-related circuitry, and specifically, GPR83 expression in the BLA may be a primary output node for regulating anxiety-related behavior.

## Data Availability Statement

The raw data supporting the conclusions of this article will be made available by the authors, without undue reservation.

## Ethics Statement

The animal study was reviewed and approved by the IACUC at Icahn School of Medicine at Mount Sinai.

## Author Contributions

AF designed, performed, and analyzed the experiments and wrote the manuscript. LL performed and analyzed the experiments and wrote the manuscript. NT performed and analyzed the experiments. LD designed the experiments and wrote the manuscript. All authors contributed to the article and approved the submitted version.

## Conflict of Interest

NT is employed by Regeneron Pharmaceuticals, Inc. The remaining authors declare that the research was conducted in the absence of any commercial or financial relationships that could be construed as a potential conflict of interest.

## Publisher’s Note

All claims expressed in this article are solely those of the authors and do not necessarily represent those of their affiliated organizations, or those of the publisher, the editors and the reviewers. Any product that may be evaluated in this article, or claim that may be made by its manufacturer, is not guaranteed or endorsed by the publisher.
